# Study of Urbach energy and Kramers–Kronig on Mn and Zn doped NiFe_2_O_4_ ferrite nanopowder for the determination of structural and optical characteristics

**DOI:** 10.1038/s41598-024-57045-7

**Published:** 2024-03-17

**Authors:** N. Nazari, M. M. Golzan, Kh. Mabhouti

**Affiliations:** Department of Physics, Faculty of Sciences, Urmia University, Urmia, Iran

**Keywords:** Mn and Zn-doped NiFe_2_O_4_ spinel ferrite, Co-precipitation method, Structural properties, Optical properties, Kramers–Kronig approach, Nanoscale materials, Structural materials, Characterization and analytical techniques, Design, synthesis and processing

## Abstract

M_x_Ni_1-x_Fe_2_O_4_ spinel ferrite (M = Mn, Zn, and x = 0, 0.05) has been successfully synthesized by co-precipitation technique with hydrazine hydrate reduction agent (instead of NaOH) and Ethylene glycol surfactant. The XRD spectra of the samples illustrated high crystallinity. The structural characterization of pure and doped fcc NiFe_2_O_4_ were calculated by Scherrer, Modified Scherrer, Williamson–Hall, and SSP methods. In comparison of several methods, the Scherrer method is unreasonable method and W–H method has an acceptable range and can calculate both < L > and strain without restriction. The specific surface area in Zn-doped increased, demonstrate increment of adsorption properties in Ni ferrite structure. TEM images revealed the shape of grains is spherical, cubic, and irregular, with a grain size in the range of 35–65 nm. Hysteresis loops illustrated the magnetic behavior of samples. From the reflectance data, the band gap energies were estimated at 1.984, 1.954, and 1.973 eV for un-doped, Mn, and Zn-doped NiFe_2_O_4_ respectively (red shift). The almost low value of Urbach energy for pure, Mn, and Zn -doped NiFe_2_O_4_ indicates low structural disorder, which can approve the high crystallinity of samples. Direct band gap energy (E_g_), refractive index, and extinction coefficient were estimated by the Kramers–Kronig method with linear optical evaluations. The E_g_ by K-K method is in good agreement with the E_g_ by Kubelka–Munk function.

## Introduction

Spinel ferrites in the general formula of AFe_2_O_4_, in which A is a divalent metal (Mg, Co, Ni, Zn, Fe, Mn, etc.), are the most widely magnetic nanoparticles (MNPs) due to their outstanding properties such as high permeability and high electrical resistivity^1,2^, and thus, have many applications^3,4^. Ferrite structure, including both M and Fe ions, can inform richer redox reactions in comparison to the single-metal oxides^2,5^. Among spinel ferrites, NiFe_2_O_4_ has specific properties such as high electrochemical stability, high permeability at high frequency, and catalytic behavior^5–7^. These features have enabled nickel ferrite semiconductors^8^ to be used in various applications such as sodium-ion batteries, gas sensors, electromagnetic wave absorption, photocatalysis, water oxidation processes, and biomedical applications^9,10^. The unit cell of spinel ferrites has eight fcc cells. It consists of 32 closely packed oxygen atoms in fcc sites and 24 metal cations distributed between 64 divalent tetrahedral (A) and 32 trivalent octahedral (B) sites. In these sites, metal cations are inscribed by four oxygen ions in tetrahedral sites and six oxygen ions in octahedral sites. NiFe_2_O_4_ spinel ferrite has an inverse spinel structure with 8 Ni^2+^ ions occupying preferentially B sites, whereas 16 Fe^3+^ ions have been equally distributed between A and B sites in the form of [Fe^3+^]^A^[Ni^2+^Fe^3+^]^B^O_4_^11^.

The cation distribution is a crucial parameter in the alteration of structural, magnetic, and optical properties of NiFe_2_O_4_ spinel ferrite. It dependent on the composition and microstructure of spinel ferrite which strongly affected by the synthesis method, synthesis conditions, and metals substitution^12^. Nickel ferrite NPs can be fabricated by using a range of wet chemical routes such as sol–gel auto-combustion^13^, hydrothermal^14^, and co-precipitation^1^. The co-precipitation method, due to its simplicity, low cost, low synthesis temperature, and size homogeneity in nano order, is widely used in the synthesis of spinel ferrites^1,15^. On the other hand, doping ferrite nanocrystals with various cations of transition metals such as Ni, Mg, Cr, Mn, Zn, Co, Cu, etc., can also alter the cation distribution in ferrites leading to a modification in their structural and optical properties^16,17^.

Mn and Zn-doped nickel ferrite can attain appealing optical features by alternating in cation distribution in A and B sites. Mn-doped NiFe_2_O_4_ has large permeability, resistivity, and small fatality compared with other dielectrics^18^. Zn-doped NiFe_2_O_4_ are specified by high surface area, high electrical resistivity, and large permeability at a high-frequency region of electronics devices (GHz)^19,20^. Aakash et al. reported electrical and dielectric reactions of transition metal ions (Zn^2+^, Cd^2+^, Mn^2+^, and Cr^3+^) and showed that the hopping length decreased by an increment in Zn, Cd, and Cr content while observed a little increment in Mn-doped nickel ferrite. They depicts all the dopant elements of NiFe_2_O_4_ have a good dielectric behavior^21^. Seema Kumari et al. synthesized Mn and Zn-doped M_x_Ni_1-x_Fe_2_O_4_ spinel ferrites using the co-precipitation method by NaOH as reduction agent and demonstrated the crystallite size of Mn and Zn doped nickel ferrite decreased to a range of 50–60 nm with an increase in the band gap energy in all dopant elements^22^. Aafiya and his coworkers prepared Zn-doped NiFe_2_O_4_ using the sol–gel auto-combustion process. They showed considerable absorbance in the visible light region and an increased direct band gap energy by Zn-doped^23^. According literature, there are almost no studies that have investigated about the structural and optical properties of Mn and Zn-doped NiFe_2_O_4_ synthesized with co-precipitation method by Hydrazine hydrate reduction agent and Ethylene glycol surfactant. Furthermore, there are no comprehensive literature in the optical properties of un-doped and Mn and Zn-doped NiFe_2_O_4_ such as Urbach energy and refractive index by DRS analysis and numerical relationships. In this study, in addition to the DRS analysis for optical characteristics, Kramers–Kronig (K-K) relations were utilized as the numerical method that is relevant to the complex part of the system to research the linear optical properties of the system and compare them to experimental results ^24^. Band gap energy, refractive index (real part), extinction (imaginary part), and absorption coefficient are the important optical parameters that can be estimated through the K-K method through the use of the DRS data.

## Experimental

### Reagents and synthesis

In this work, Iron (III) chloride hexahydrate (FeCl_3_·6H_2_O, 98%, Merck), Nickel chloride hexahydrate (NiCl_2_.6H_2_O, 98% Merck), Manganese (II) chloride dihydrate (MnCl_2_·2H_2_O, 98%, Merck), Zinc (II) chloride (ZnCl_2_, 98%, Merck), Ethylene glycol (Merck) and Hydrazine hydrate (NH_2_.NH_2_.H_2_O) (Merck 100%), were used without any further purification.

NiFe_2_O_4_ and M_0.05_Ni_0.95_Fe_2_O_4_ (M = Mn, Zn) nanoparticles were synthesized by the chemical co-precipitation technique. 0.1 M of nickel chloride and 0.2 M of iron chloride solutions with stoichiometric ratios Ni:Fe = 1:2, were solved separately in deionized water. The as-prepared solutions were mixed and stirred intensely for 20 min to obtain a homogenous mixture. Ethylene glycol was added into the solution as a surfactant with stoichiometric ratios surfactant: metals = 0.5:1, and mixed at 60 °C. Surfactant disturb the fixity of the mixed solution and makes the electrostatic repulsion force that can prevent the clumping of particles in the synthesis process and help control crystallite size^25^. Subsequently, a suitable amount of hydrazine hydrate as a reduction agent was added dropwise into the solutions under vigorous stirring, and black color precipitates formed in the pH = 10 and the nucleation of nanoparticles initializes. Hydrazine hydrate can control the PH of the reaction and so decreases the clumping of particles to control the particle size without any impurities in the reaction mixture^26^. The prepared slurry was kept under vigorous magnetic stirring for 1:45 min at 80 °C and then was cooled to RT. The precipitate was separated through the strong magnet, rinsed several times by water and ethanol to get the neutral PH, and dried in a hot air oven for 24h at 60 °C. Finally, the grinding powder was annealed at 800 °C for 2 h in a muffle furnace. The annealing temperature and time are essential factors in synthesized nanoparticles because, the initialization of phase transition processes and, consequently, the formation of a spinel structure of the NiFe_2_O_4_ begin at this temperature. Also, in high annealing temperatures, the structural defects that are due to synthesis at lower annealing temperatures, are improved^26^. The above procedure was repeated to prepare of Mn and Zn-doped NiFe_2_O_4_ NPs by adding manganese chloride and zinc chloride in stoichiometric ratios M:Ni:Fe = 0.05:0.95:2, in the solution.

### Characterization and measurements

Structure determination of the specimens characterized via X-ray diffraction (XRD) patterns by using Philips PW 1730 diffractometer with Cu-Kα radiation (λ = 1.54056Å) in the 2θ = 10–80 degrees and patters were plotted by the X’Pert high score plus software. The microstructural characterizations of NPs were recorded by the transmission electron microscopy (TEM) analysis set (Philips EM 208S Netherland). The magnetic characteristics of the samples was measured using a vibrating sample magnetometer (VSM-MDKB) in the 1.4 T field at room temperature. The optical properties were measured through the diffuse reflectance spectroscopy (DRS) measurement (Sinco S4100 Korea). The linear optical calculations were done by MATLAB coding.

## Result and discussion

### Structural analysis

**Figure 1:**
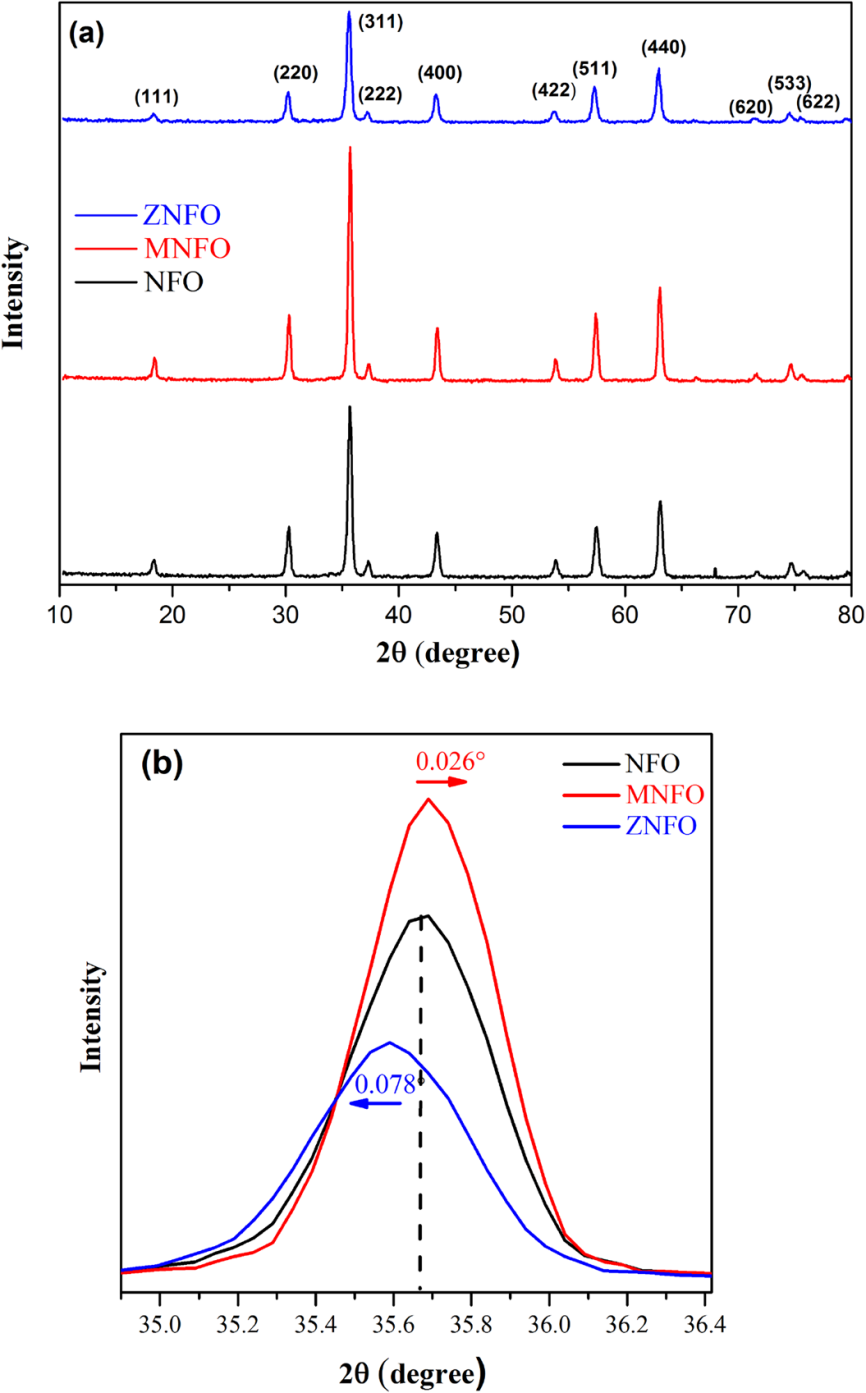
(**a**) XRD patterns, (**b**) Enlarge the view of the most intense peaks in (311) plane for un-doped, Mn-doped, and Zn-doped NiFe_2_O_4_.

XRD patterns of NiFe_2_O_4_, Mn, and Zn-doped NiFe_2_O_4_ nanopowders which were annealed at 800 °C and named NFO, MNFO, and ZNFO, respectively, are depicted in Fig. 1a. It can be observed that all the appearance diffraction peaks for NFO, MNFO, and ZNFO spinel ferrites can have perfect condition indexed to reflection at (111), (220), (311), (222), (400), (422), (511), (440), (620), (533) and (622) crystal planes of NiFe_2_O_4_ inverse spinel structure with the cubic spinel structure (fcc) and Fd3m space group that have an accommodation with the standard spectrum JCPDS Card (No. 96-591-0065). The sharp peaks in the patterns of specimens can denote that the structure of samples has a high crystallinity phase in the inverse spinel structure of NiFe_2_O_4_. It can be associated with heat treatment, in high annealing temperature, the impurities eliminate, and the stress of the unit cell diminishes, hence atoms appoint more appropriately at the suitable lattice sites so the crystallinity increases and single crystal form instead of polycrystal structure, so more substantial Bragg’s diffraction apparent^27^. Also use of hydrazine hydrate can diminish the molesting phase. From Fig. 1a, the XRD pattern in NFO demonstrates a weak impurity peak in 2θ = 67.95°, which can be due to holder contamination of the XRD device in the laboratory. XRD pattern of MNFO in Fig. 1a shows no impurity phase or extra diffraction peaks related to Mn oxides or Mn impurity or clusters. Also, the pattern depicts that the diffraction peak intensities increased and peaks wide decreased in the Mn-doped compared to un-doped nickel ferrite, that this can imply the highest crystallinity and increment in crystallite size of MNFO. A bit shift in the position of the diffraction peaks of MNFO and ZNFO patterns in Fig. 1a can be attributed to the distinction in dopant ionic radii^22,27^ that is shown in Fig. 1b for the (311) plan. ZNFO spectra in Fig. 1a indicate no new crystalline phases corresponding to secondary phases, ZnO, Zn, or impurity phases in the structure of ZNFO spinel ferrite. The broadening and low-intensity peaks of ZNFO can imply a decrement in crystallinity and crystallite size despite the MNFO. According to the above mentioned, it can result that Mn and Zn doped ions are entirely incorporated in ions sites of NiFe_2_O_4_ structure and have a different effect in structural properties. Also, from the Fig. 1a, it is evident that all XRD patterns of synthesized samples demonstrated the diffraction peak intensity of the (311) crystal plane was the highest that indicating the preferential orientation growing of NiFe_2_O_4_ crystal structure is along the (311) crystal plane^23,27^. The peak positions (2θ) of the synthesized samples were estimated by using X’Pert High Score Plus software and tabulated in Table 1.

**Table 1:** Peak positions of X-ray diffraction (2θ) of the NFO, MNFO, and ZNFO spinel ferrites.

Peak no	hkl	NFO	MNFO	ZNFO
		2θ (°)	2θ (°)	2θ (°)
1	(111)	18.304	18.385	18.29
2	(220)	30.222	30.259	30.172
3	(311)	35.635	35.661	35.557
4	(222)	37.273	37.299	37.19
5	(400)	43.342	43.367	43.256
6	(422)	53.828	53.828	53.68
7	(511)	57.414	57.369	57.241
8	(440)	63.052	63.023	62.887
9	(620)	71.61	71.53	71.40
10	(533)	74.636	74.580	74.463
11	(622)	75.67	75.56	75.471

#### Lattice parameter, x-ray density, and hopping length

The lattice parameter (a) and cell volume (V) can be computed from the XRD patterns by the following equations by X’Pert High Score Plus software:

1 $${d}_{hkl}=\frac{a}{\sqrt{{h}^{2}+{l}^{2}+{k}^{2}}},$$

2 $$a=d\sqrt{{h}^{2}+{l}^{2}+{k}^{2}},$$

3 $${V}_{cell}= {a}^{3}.$$

d_hkl_ is the inter-planer distance in h, k, l miller indices, as can be seen from Table 2, the lattice parameter in MNFO and ZNFO increased in comparison to NFO, which can be associated with the cation distribution. When spinel ferrite is doped, the dopant ion is generally replaced in the sites that are proportional to ionic radii^28^. In Mn-doped NiFe_2_O_4_, with regarding MnFe_2_O_4_ as partial inverse spinel ferrite with 80% Mn^2+^ at tetrahedral and 20% Mn^2+^ at octahedral sites, because of larger ionic radius of Mn^2+^ (0.66 Å) to Fe^2+^ (0.49 Å) ions at A sites and larger ionic radius of Mn^2+^ (0.83 Å) to Ni^2+^ (0.69 Å) ions at B sites of inverse spinel NiFe_2_O_4_, the lattice parameter increased, and the unit cell can be expanded. Also, in the Zn-doped NiFe_2_O_4_, the Zn^2+^ ions have a preference to present in the tetrahedral sites with larger ionic radius (0.65 Å) to Fe^2+^ (0.49 Å) ions in A sublattice hence the lattice constant in ZNFO can be larger than NFO^29,30^. The amount of x-ray density (ρ_x_) is estimated by the following relation:

4 $${\rho }_{x}=\frac{ZM}{{N}_{A}{a}^{3}}= \frac{8M}{{N}_{A}{a}^{3}} ,$$

where M is the molecular weight, Z = 8 is the number of molecules per unit cell, and N = 6.022 × 10^23^ (atoms/mol) is the Avogadro number. From Table 2, it can be seen that the ${\rho }_{x}$ decreased in Mn and Zn-doped NFO. This can be due to the decreasing surface tension that was created by the lattice constant, the size effect, and the expansion of the unit cell by replacing Zn^+2^ or Mn^+2^ ions instead of Ni^+2^ ions^1^.

**Table 2:** Crystallographic parameters of the NFO, MNFO, and ZNFO spinel ferrites.

Samples	Crystal system	lattice constant a (Å)	Unit cell volume V (Å^3^)	x-ray density ${\rho }_{x}( gr/{cm}^{3})$	Hopping length	
					L_A_ (Å)	L_B_ (Å)
NFO	FCC cubic	8.3326	578.5509	5.3817	3.6081	2.9460
MNFO	FCC cubic	8.3373	579.5305	5.3683	3.6101	2.9476
ZNFO	FCC cubic	8.3531	582.8315	5.3498	3.6169	2.9532

The hopping length is the parameter that gives information about the distance between magnetic ions and the strength of the spin interaction of ions that is defined as the following equations^31^:

5 $${L}_{A}=\frac{a\sqrt{3}}{4} ,$$

6 $${L}_{B}=\frac{a\sqrt{2}}{4},$$

where *L*_A_ is the hopping length at the A site (the shortest interval between the A site and the oxygen atom), and *L*_B_ is the hopping length at the B site (the shortest interval between the B site and the oxygen atom)^32^. As can be seen in Table 2, L_A_ and L_B_ increased in MNFO and ZNFO compared to NFO. This increase can be due to the alteration in ionic radii in the A and B sites by the insertion of Mn^2+^ and Zn^2+^ dopant ions as explained in the lattice constant alteration section, so this induces increment in lattice constant, and unit cell and the hopping length between magnetic ions in Mn and Zn doped NiFe_2_O_4_ increased.

#### Crystallite size

The crystallite size is the parameter that has a crucial role in all properties of MNPs, so in this research, to the precise calculation of the crystallite size and strain of the nanopowders, four essential methods have been used: Debye–Scherrer, Modified Debye–Scherrer, Williamson-Hall, and the Size Strain plot methods. The full width at half maximum (FWHM) of all peaks position has been estimated by X’Pert high score plus software and fitted with this software. The peaks broadening of diffraction patterns can be due to crystallite size and local lattice strain that both are physical broadenings, and also by instrumental broadening^31^. To calculate the precise crystallite size value of nanopowder, the instrumental broadening must be deleted from observed broadening, so according to Marzieh Rabiei et al.^33^ and M. A. Islam et al.^31^ the following equation represents the refined broadening:

7 $${{\beta }_{d}}^{2}={{\beta }_{o}}^{2}-{{\beta }_{i}}^{2} \Rightarrow {\beta }_{d}=\sqrt{{{\beta }_{o}}^{2}-{{\beta }_{i}}^{2}} .$$

In this relation, β_i_ is the instrumental broadening, β_o_ is the observed broadening, and β_d_ is the reformed broadening that was used to calculate crystallite size. In many articles, broadening of crystalline silicon is used as instrumental broadening (β_instrumental_ = β_standard_ = 0.196 degrees) for calibration and removal from the observed peak width^33^.

##### Debye–Scherrer method

The Scherrer method was generally used to calculate crystal size for the most intense peak, which is depicted as follows^32^:

8 $$L= \frac{K\lambda }{\beta }\cdot \frac{1}{cos\theta } ,$$

Here, L is the crystallite size, K is the structure factor that usually takes as 0.9, λ is the wavelength of the X-rays source (λ = 0.15405 nm), θ is the braggˊs angle of the peak, and β is the full width at half maximum (FWHM) of the most intense diffraction peak. To compute the average crystallite size, the most substantial (311) peak was used in the Sherrer equation, and the estimated value was tabulated in Table 3.

**Table 3:** Calculated values of average crystallite size < L > and specific surface area (SSA) values for NFO, MNFO, and ZNFO spinel ferrites.

Samples		Debye Sherrer		Modified Debye Sherrer		Williamson–Hall		Size strain plot	
		< L > (nm)	(SSA)$\frac{{m}^{2}}{gr}$	< L > (nm)	(SSA)$\frac{{m}^{2}}{gr}$	< L > (nm)	(SSA)$\frac{{m}^{2}}{gr}$	< L > (nm)	(SSA)$\frac{{m}^{2}}{gr}$
NFO		23.56	47,321	25.73	43,330	23.69	47,061	25.20	44,241
MNFO		27.45	40,716	31.68	35,280	30.59	36,537	34.57	32,330
ZNFO		19.92	56,302	22.17	50,588	16.04	69,921	15.33	73,159

##### Modified Debye–Scherrer (Monshi–Scherrer) method

In the Scherrer method, nanocrystalline size L was calculated for the most intense peak and supposed that crystallite size must be a constant value for different peaks of sample. In contrast, by increasing of 2θ value, the amount of cos θ is not retained constant, so caused an error in the L value^33^. Monshi et al. in 2012^34^ used the Modified Scherrer method to decreasing the errors or $\sum (\pm {\Delta ln\beta )}^{2}$ to calculate a more precise value of nanocrystalline size and was estimated by making logarithm on both sides of the Scherrer equation^33^:

9 $$ln\beta =ln\frac{K\lambda }{L\cdot cos\theta }=ln\frac{K\lambda }{L}+ln\frac{1}{cos\theta }.$$

**Figure 2:**
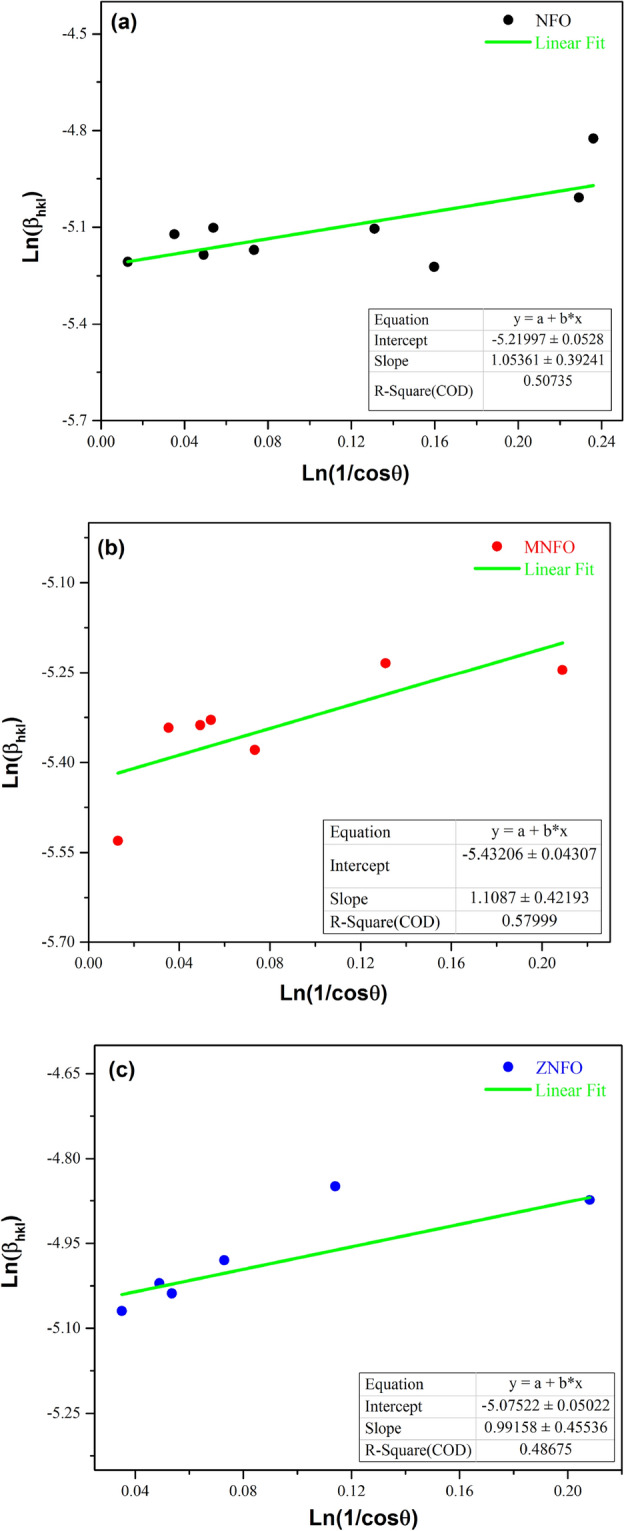
Modified Debye-Scherer plots for the synthesized samples: (**a**) NiFe_2_O_4_, (**b**) Mn-doped NiFe_2_O_4_, (**c**) Zn-doped NiFe_2_O_4_.

The average value of crystallite size is calculated by plotting Ln β (radians) versus Ln(1/Cosθ) for all the selected peaks as y = ax + b equation as shown in Fig. 2 and Ln (Kλ/L), or y-intercept is approximated by the linear fitting of the plot through the origin software^31^. The fixed L value is determined by the following equation:

10 $$\frac{K\lambda }{L}={e}^{(intercept)}.$$

In addition, in the Monshi–Scherrer method, the slope of the plot must be one, therefore, some of the peak points must be deleted from the plot to achieve the slop of almost one, as can be seen in Fig. 2. but for the other next two methods, the points of plots weren’t deleted to the appropriate comparison between methods. The calculated data are presented in Table 3.

##### Williamson-Hall method

In the Scherrer and Modified Sherrer method, the XRD peaks broadening only arises from the crystallite size and cannot be considered the microstructures of the lattice and intrinsic strain effect, therefore, Scherrer and Modified Scherrer methods can only determine the crystallite size, but Williamson–Hall method considers the strain in addition of the crystallite size in peak broadening of all crystallographic orientations and estimate both L and strain ε value of the crystal structure. A total physical broadening (b_tot_) has been estimated by the following equations^35^:

11 $${\beta }_{tot}={\beta }_{hkl}={\beta }_{strain}+{\beta }_{crystal}=4\varepsilon\, tan\theta +\frac{0\cdot 9\lambda }{Lcos\theta },$$

12 $${\beta }_{hkl}cos\theta =4\varepsilon sin\theta +\frac{0\cdot 9\lambda }{L }.$$

**Figure 3:**
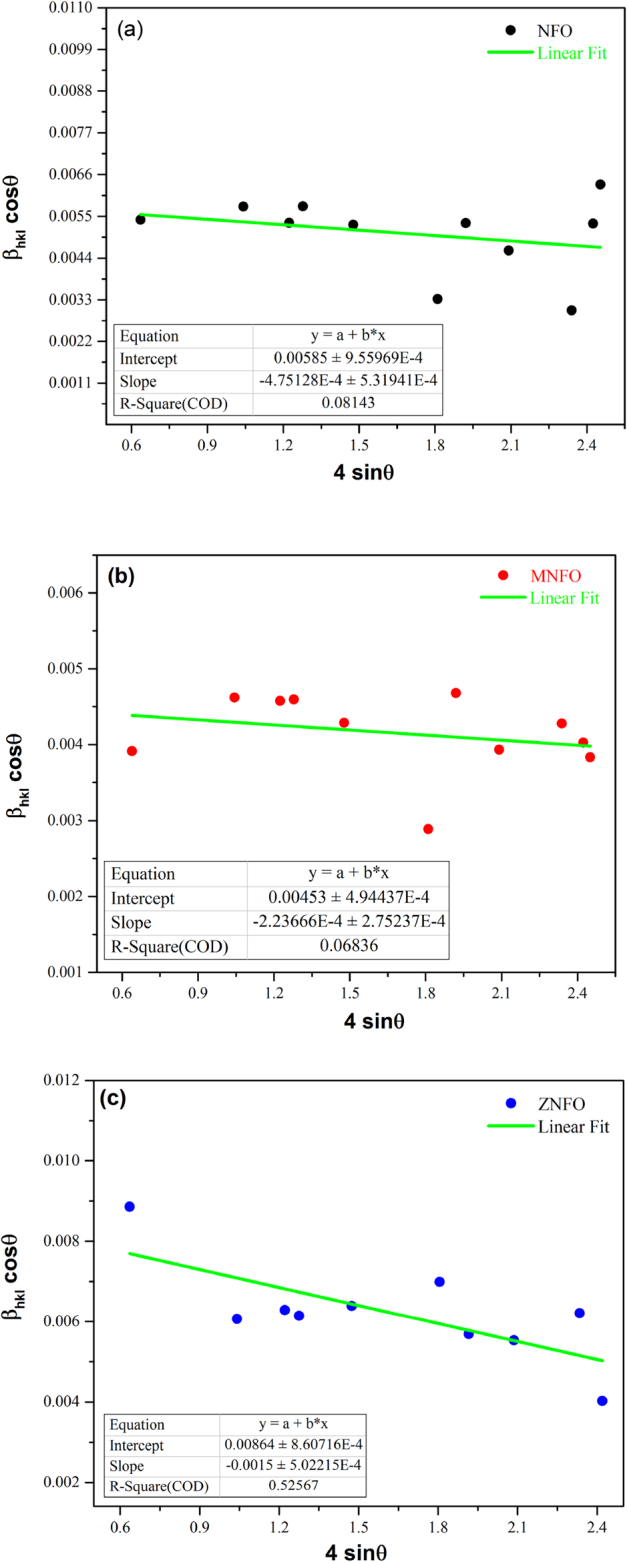
Williamson–Hall plots for the synthesized samples: (**a**) NiFe_2_O_4_, (**b**) Mn-doped NiFe_2_O_4_, (**c**) Zn-doped NiFe_2_O_4_.

Figure 3 shows the b_hkl_ cosθ plot drawn against 4 sinθ by XRD data for all samples. The linear fitting in this graph was done by origin software, and the slope and y-intercept of the graph were specified, and the ε and L values tabulated in Tables 3 and 4.

**Table 4:** Calculated values of lattice strain and micro-strain for NFO, MNFO, and ZNFO spinel ferrites.

Samples	Lattice strain by formula (ε = $$\frac{\beta }{4tan\theta }$$)	Micro-strain by formula (ε = $$\frac{\beta cos\theta }{4}$$)	W–H micro-strain (ε = $$slope)$$	SSP micro-strain (ε = 2 $$\sqrt{slope}$$)
FO	0.00357	0.00126	– 0.00047	–
MNFO	0.00288	0.00103	– 0.00022	0.0043
ZNFO	0.00478	0.00155	– 0.0015	–

##### Size–Strain Plot method (SSP)

**Figure 4:**
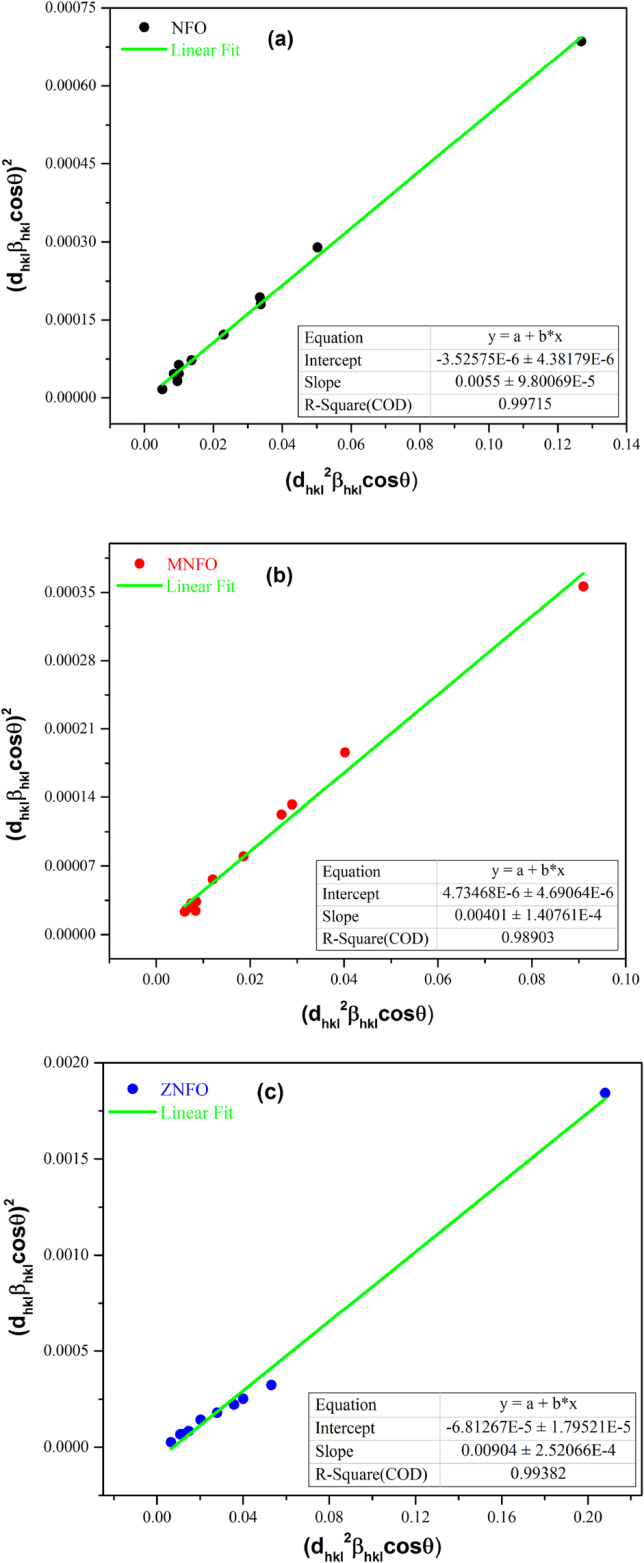
Size–Strain plot (SSP) for all samples: (**a**) NiFe_2_O_4_, (**b**) Mn-doped NiFe_2_O_4_, (**c**) Zn-doped NiFe_2_O_4_.

**Figure 5:**
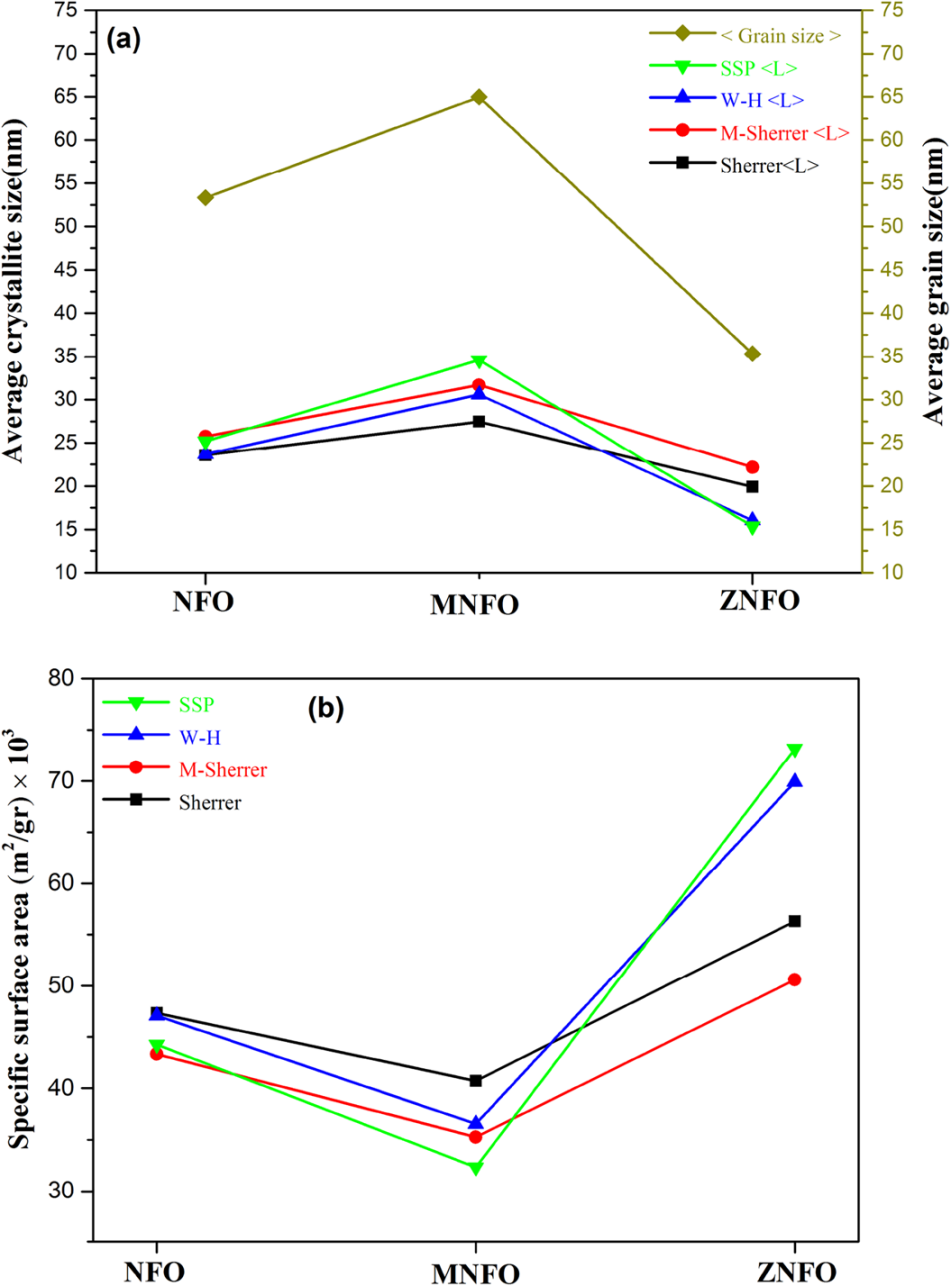
Plots of variation, (**a**) average crystallite size, (**b**) specific surface area.

In this method, lower-angle reflections are more important than higher-angle reflections. Because at higher angles of diffraction peaks, the data of the XRD pattern has less modality, and diffraction peaks have an overlap. Functions used in the SSP method are the Gaussian and Lorentzian functions, which describe the strain and size profile of crystalline materials, respectively. The total broadening of the SSP method is defined as follows^33^:

13 $${\beta }_{hkl}={\beta }_{L}+{\beta }_{G},$$

where β_L_ and β_G_ are the peaks broadening, which have been derived from Lorentz and Gaussian functions, the SSP method is expressed through the following equation^33^:

14 $${({d}_{hkl}{\beta }_{hkl}cos\theta )}^{2}=\frac{K\lambda }{L}\left({d}_{hkl}^{2}{\beta }_{hkl}cos\theta \right)+\frac{{\varepsilon }^{2}}{4},$$

where d_hkl_ is the lattice distance for different (hkl) planes. To calculate the size and strain of nanoparticles, the plot was drawn with ${({d}_{hkl}{\beta }_{hkl}cos\theta )}^{2}$ as a y-axis and ${{d}_{hkl}}^{2}{b}_{hkl}cos\theta$ as x-axis and y = ax + b as linear fit to experimental data by using the origin software as exhibited in Fig. 4. The values of L and ε were tabulated in Table 3 and Table 4. However, in the case of strain value, because of the negative intercept value for NFO and ZNFO, the strain value cannot be calculated. Figure 5a illustrates the compares of average crystallite size by all used methods for all specimens.

In comparison methods of the calculating crystallite size, Scherrer method is unreasonable method but other methods have acceptable range according literature and among them, Williamson-Hall method can be estimated both size and strain values in acceptable range without some restrictions that exist in M-Scherrer and SSP methods as explained. From the calculating crystallite size for synthesized MNPs, according to Table 3 and Fig. 5a, the crystallite size of MNFO increased compared to NFO MNPs. It can be explained that Mn^2+^ dopant cations have stronger Mn^2+^-o^2–^ bonds in comparison to Ni^2+^-o^2–^ bonds, so this can cause the crystallite size increased. Another reason can be attributed to the electronic configuration of Mn^2+^ (3d^5^) in comparison to Ni^2+^(3d^8^). Mn^2+^ ions have more electrons than Ni^2+^ ions, hence Mn^2+^ ions attitude to more covalent interaction with its ligand and oxygen anions compared to Ni^2+^ ions so that, these factors can result in increasing the crystallite size of MNFO spinel ferrite^2^.

According to Table 3 and Fig. 5a, the decrement in crystallite size of ZNFO, at first, can be attributed to the cation distribution of the ZNFO structure. Bulk NiFe_2_O_4_ has an inverse spinel structure, however, in the nano-sized NiFe_2_O_4_, the spinel structure can have a mixed spinel structure for the size smaller than a few nm or by doping^11^ that in this structure, divalent cations distributed in both A and B sites [Fe _x_^3+^M_1-x_^2+^]^A^[M_x_^2+^Fe _2-x_^3+^]^B^O_4_. In the ZNFO structure, Zn^2+^ ions strongly prefer to be in the tetrahedral site of NiFe_2_O_4_ structure and caused mixed spinel structure, so this can cause disorder in the favorable Fe^3+^ ions site in tetrahedral sublattice and this excluded the growth of grains^16,30^. Second, it can be related to the complete electronic configuration of the Zn^2+^ ion (3d^10^) and lake of interaction electrons that resulted in fewer interactions with its ligands and oxygen ions, so crystallite size decreased. The third reason can be related to the lower bond energy of Zn^2+^-O^2–^ (159 kJ/mol) compared to Ni^2+^-O^2-^ bond energy (451 kJ/mol) which causes a decrement in crystallite size^20^.

#### Specific surface area

In the materialˈs structure, the specific surface area (SSA) can be specified physical and chemical processes of materials and is the overcoming variable for adsorption applications of nanoparticles, which is expressed by the following equation^36^:

15 $$S=\frac{6000}{{\rho }_{x}\times L},$$

where S is the specific surface area, which has an inverse relation with crystallite size and X-ray density. Table 3 depicts the SSA values via crystallite size estimated by the four mentioned methods for all samples. As can be seen, the specific surface area was reduced by Mn-doped NiFe_2_O_4_ because of an increment in crystallite size, whereas by Zn-doped NiFe_2_O_4_, the specific surface area increased due to decreasing crystallite size. The high specific surface area of synthesized nanopowders would assist the adsorption properties by incrementing of the active sites of NiFe_2_O_4_ nanoparticles for catalyst applications^22,37^. Figure 5b demonstrates variations of the specific surface area calculated by four methods.

#### Lattice strain and micro-strain

In the NiFe_2_O_4_ structure, oxygen atomsˊ positions in tetrahedral and octahedral sites mean: FeO_6_, NiO_6_, and FeO_4_ can be changed by Mn^2+^ and Zn^2+^ doped ions, and this highly disturbs the lattice and affect physical and chemical properties, especially structural parameters such as strain, dislocation density, stacking fault coefficient and packing factor^6^.

In the crystalline structure, interstitial atoms and lattice dislocations can induce pressure on the atoms and slightly stray them from their usual positions. This pressure is defined as lattice strain (ε_ls_), which can deform the unit length in the crystal structure. This parameter is calculated by the following equation^38^:

16 $${\varepsilon }_{ls}=\frac{\beta }{4tan\theta },$$

The deformation of atoms in the crystal structure, which can be due to displacement, domain boundary defects, and point defects, occurs in one per million parts of the materials and is called the micro-strain (ε_ms_) that is defined by the following equation^38^:

17 $${\varepsilon }_{ms}=\frac{\beta cos\theta }{4}.$$

**Figure 6:**
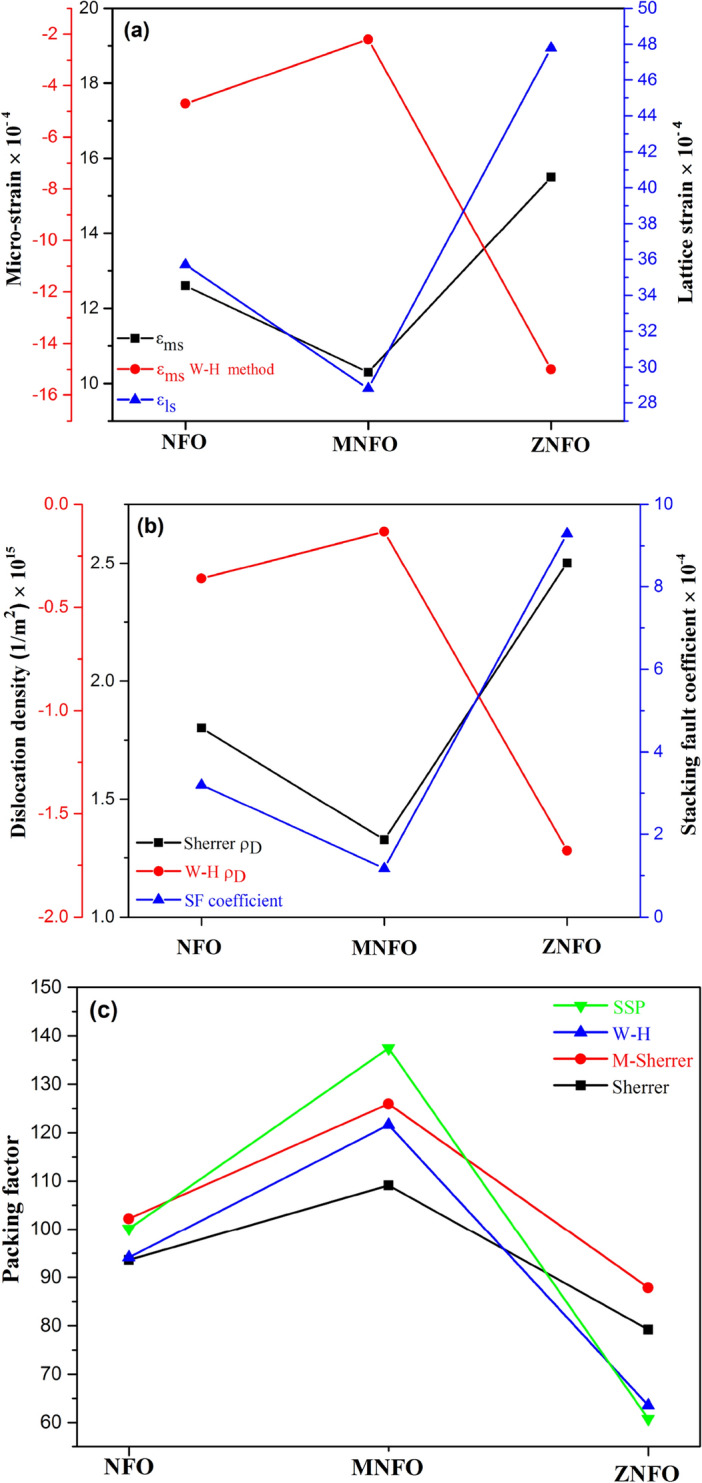
Plots of variation (**a**) Micro strain, (**b**) dislocation density, (**c**) Packing factor for NFO, MNFO, and ZNFO spinel ferrites.

Table 4 depicts the strain values for all samples. As can be seen, the strain values of MNFO decreased, and the strain of ZNFO increased in comparison to NFO, which can be a result of the crystallinity of XRD patterns in Fig. 1a. Furthermore, the structural parameters such as dislocation density and stacking fault coefficient are impressive in the strain value of synthesized nanopowders. It should be noted that the negative value of the micro-strain in the Williamson-Hall method implies a compressive strain in the samples. It can be due to the different ions with different ionic radii, including Ni^2+^, Fe^3+^, Mn^2+^, and Zn^2+^, in the structures of samples^1,39^. Variations of micro-strain and lattice strain are shown in Fig. 6a.

#### Dislocation density and stacking fault coefficient

Dislocation density is the parameter that demonstrates irregularity within a crystal structure and specifies the number of dislocation lines length per unit volume of the crystal structure and defined with the following equations^40^:

18 $${\rho }_{D}=\frac{1}{{L}^{2}} \mathrm{sherrer\, method},$$

19 $${\rho }_{D}=\frac{15\varepsilon }{aL} {\text{Williamson}}-\mathrm{Hall\, method}.$$

The negative value of dislocation density by the Williamson-Hall method can be due to the discretion of chosen dislocation basis, which is represented in Table 5^41^.

**Table 5:** Calculated values of dislocation density (${\rho }_{D})$, packing factor (P), and stacking fault coefficient for NFO, MNFO, and ZNFO spinel ferrites.

Samples	Debye-Sherrer		Modified Debye-Sherrer	Williamson-Hall		Size strain lot	SF in (311) peak
	${\rho }_{D} (1/{m}^{2}$)	P	P	${\rho }_{D}(1/{m}^{2})$	P	P	
NFO	18.01 $\times {10}^{14}$	93.59	102.22	– 3.609 $\times {10}^{14}$	94.11	100.14	0.00032
MNFO	13.26 $\times {10}^{14}$	109.128	125.94	− 1.323 $\times {10}^{14}$	121.62	137.44	0.000117
ZNFO	25.01 $\times {10}^{14}$	79.24	87.88	− 16.791 $\times {10}^{14}$	63.58	60.79	0.000929

In the fcc structure of spinel ferrite, the stacking sequence is the form of ABCABCABC layers. When any kind of perturbation happens in this sequence, the stacking fault appears and can shift the peaks position of nanoparticles compared to order reference spectra peaks^32^. This coefficient (SF) is determined by the following equation:

20 $$SF= \left[\frac{2{\pi }^{2}}{45{(3tan\theta )}^\frac{1}{2}}\right]\Delta \left(2\uptheta \right),$$

where θ is the Bragg’s angle, which here, the Bragg’s angle of the strongest peak (311) regarded to calculating SF coefficient, and $\Delta 2\theta$ is the difference in reference and observed 2θ values^40^. As present in Table 5, the stacking faults are shallow values in order of 0.0001, that this depicts that the positions of observed peaks are very close to the expected values, and the bit-shifted values can be related to created disorder by Mn^2+^ and Zn^2+^ dopant agents. The SF value of MNFO and ZNFO respectively decreased and increased in compare NFO. The decrement SF parameter in MNFO conforms to the reduction of disorder and increasing crystallinity that evident from the decreasing of peak wide and increasing intensity at (311) peak in XRD section. In contrast, increasing SF value in ZNFO depicts decreasing crystallinity and increment in the defect that can be estimated from larger peak wide and lower peak intensity. Alterations of dislocation density and stacking fault coefficient are illustrated in Fig. 6b.

#### Packing factor

The packing factor is determined from the following relation:

21 $$P=\frac{L}{d},$$

L is the particle size, and d is inter-planer spacing^29^. The values of the packing factor for all samples are represented in Table 5 and illustrated in Fig. 6c.

### Microstructural analysis

**Figure 7:**
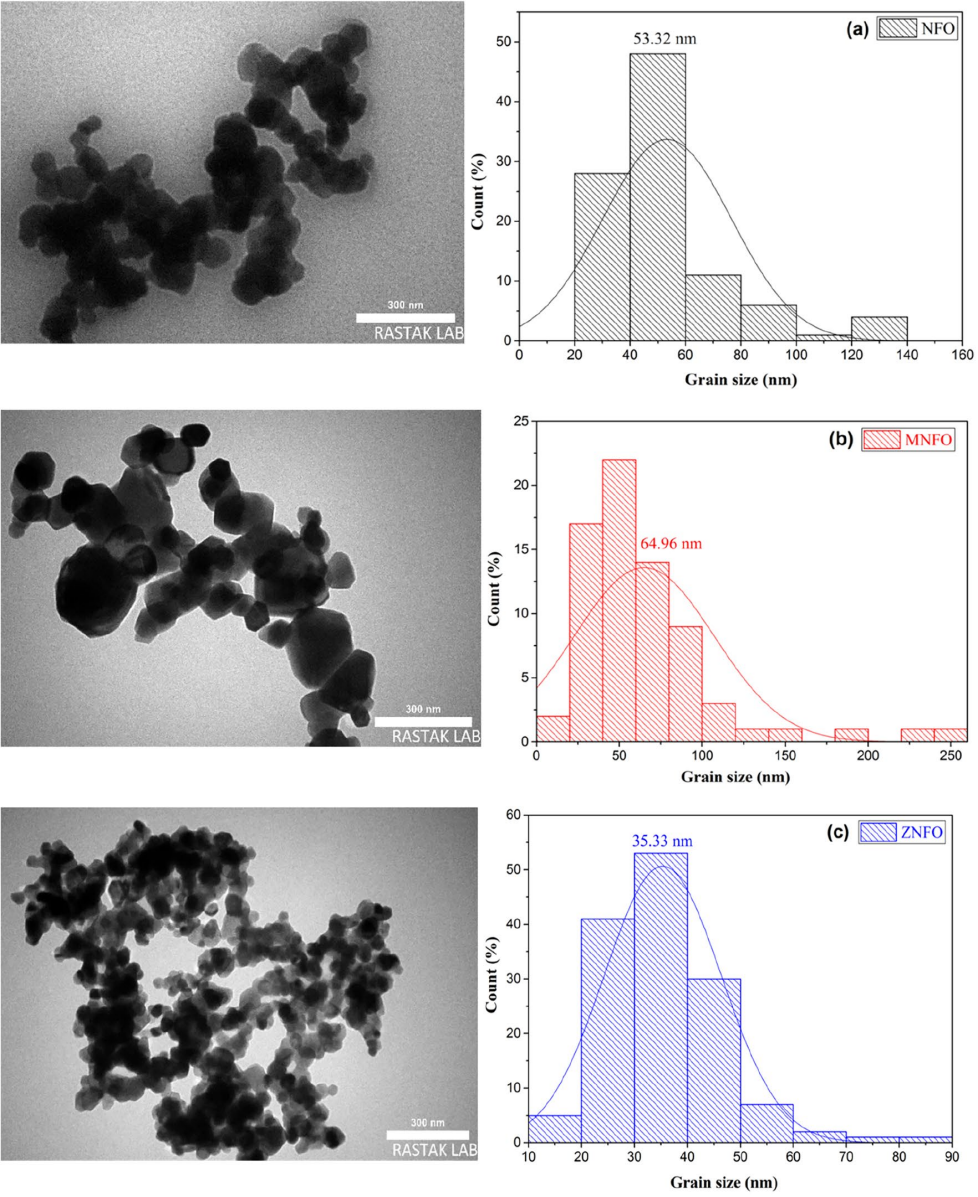
TEM images and grains size distribution histogram with Gaussian fit of (**a**) NFO, (**b**) MNFO, and (**c**) ZNFO spinel ferrites.

Figure 7 shows the TEM images of three samples of NiFe_2_O_4_ ferrites. As can be seen, the synthesized MNPs are high crystallized because of heat treatment, at high annealing temperature (800 °C), the lattice strain broadening decreased by declining or eliminating contamination species through the diffusion of tiny grains (impurity) into the bigger ones (Ostwald ripening procedure) and microstructures of spinel ferrites were refined^29^. Also, Fig. 7 demonstrates that the shape of MNPs in NFO, MNFO, and ZNFO is almost both spherical and cubic type with a bigger and smaller size, and also irregular shapes for the other grains, which are separated by a grain boundary. Irregular shapes can arise from the first, agglomeration of the particles^42^, which can be because of first, heat treatment (Ostwald ripening process), and the second, magnetic interaction between particles that arise from the magnetic nature of some components such as Fe, Ni, and Mn in the structure of spinel ferrites^42^.

Wide particles sizes distribution was estimated by histogram distribution as shown in Fig. 7. The grains size of NFO range from 21 to 138 nm for 98 MNPs, the MNFO sample range from 17.06 to 246.06 nm for 72 MNPs, and the ZNFO sample range from 14 to 85 nm for 140 MNPs. Average grains sizes were obtained at 53.32 nm, 64.96, and 35.33 nm for NFO, MNFO, and ZNFO, respectively. As can be seen, the average grains size estimated for all tree samples by TEM images are more significant than their respective average crystallites sizes that were determined by XRD analysis, which illustrates in Fig. 5a. This difference can arise from the high ratio of surface area to volume which cause to gathering of several crystallite size to form bigger grain size also other reason can be related to agglomeration in high calcination temperature to earn a minimum of Gibbs energy^43^. However, in comparing the average grains size of NFO, MNFO, and ZNFO to each other, there is a good agreement whit XRD results. The difference in the average particles size between doped and un-doped nanopowders can be attributed to metal oxides’ formation reaction in various kinetics in ferrite structure or arise from the diverse ionic radius of metal ions that induced strain in the lattice structure^27^.

### Magnetic properties

**Figure 8:**
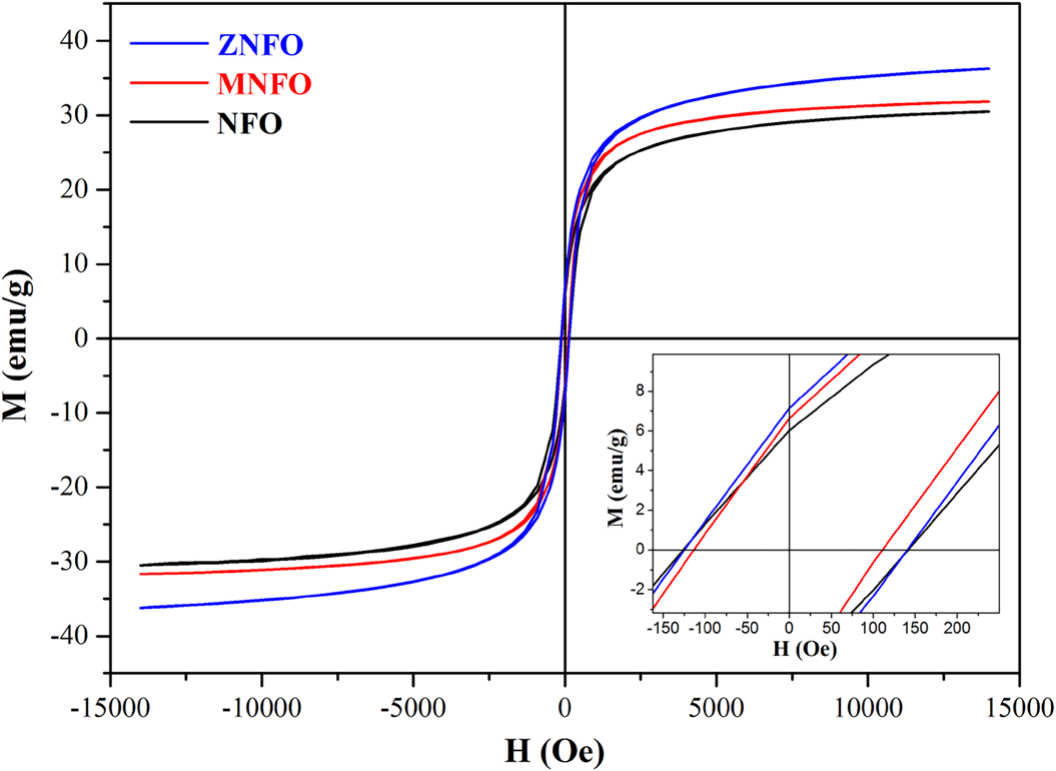
Magnetic hysteresis loops of pure and Mn, Zn-doped Ni ferrites nanoparticles.

Hysteresis loops of the NiFe_2_O_4_ MNPs were obtaid by the vibrating sample magnetometer (VSM) at 300 K, as depicted in Fig. 8. *M*_s_ values of NFO, MNFO, and ZNFO nanoparticles were represented 30.49 emu/g, 31.84 emu/g, and 36.25 emu/g respectively. Furthermore, the Coercivity fields of hysteresis loops for NFO, MNFO, and ZNFO illustrated the values 126.15 Oe, 113.55 Oe, and 124.67 Oe, respectively.

### Optical properties

#### Reflectance

**Figure 9:**
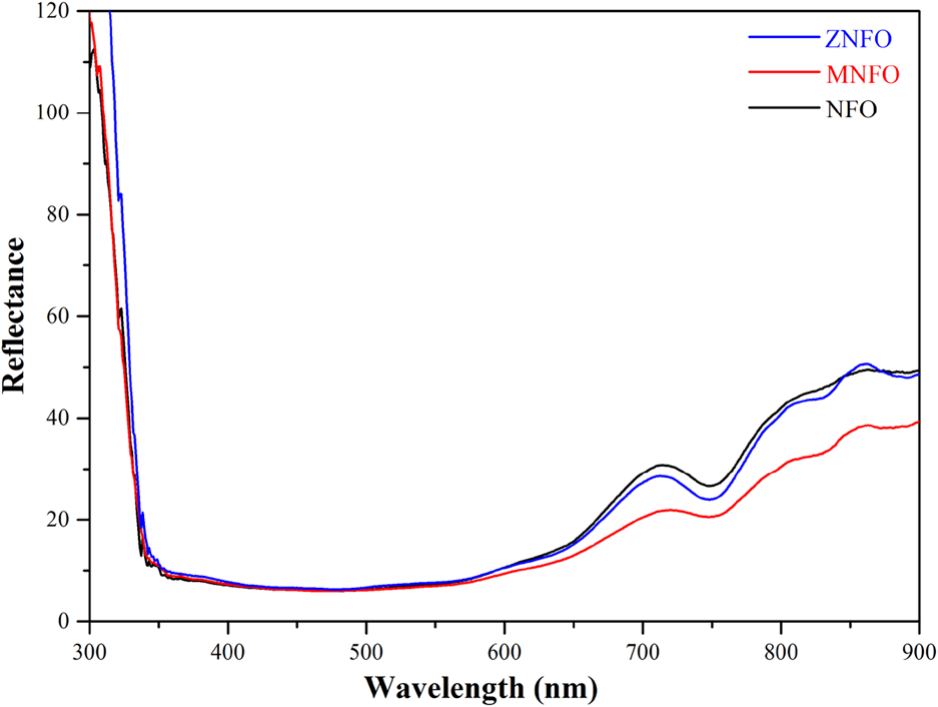
DRS spectrum of the samples at room temperature.

Electronic interactions and optical characterization of the nanoparticles were investigated by diffused reflectance spectroscopy (DRS) in the UV–visible region. In this region, the hardship of getting absorption due to enhanced scattering is deleted. Figure 9 demonstrates the reflectance spectra of the synthesized specimens in the range of λ = 300–900 nm at room temperature. As it can be seen, in λ < 350 nm, there is a sharp decrement in reflectance spectra and a broad dip in the range of λ = 350–600 nm, which are in the UV and visible light region that achieve minimum value in about λ = 476 nm for NFO, MNFO, and ZNFO spinel ferrites. The reflectance peak around λ = 714 nm in the visible region has a bit shift to a higher wavelength in Mn-doped spectra. Accordance Fig. 9, the NFO spinel ferrite depicted a greater reflectance at the range of about 600–800 nm in the visible region compared to the MNFO and ZNFO spinel ferrites, which can be implied a reduction of reflectance in Mn and Zn-doped nickel ferrite in the visible region. From Fig. 9, in the reflectance spectra of MNFO, the minimum reflectance around 750 nm in the visible region slightly shifted to a higher wavelength, that this can be due to various optically active sub-levels that conformed intranet the band gap of MNFO. In the near-IR ranges (λ > 800 nm), the reflectance increased in compared to the visible region.

##### Kubelka–Munk function

The optical band gap (E_g_) is the main parameter in optoelectronic applications. To calculate E_g_, the Kubelka–Munk function (F(R)) is utilized by absorption spectra from DRS data that is defined as the following relation^44,45^:

22 $${\text{F}}\left({R}_{\infty }\right)\propto \frac{{\left(1-{R}_{\infty }\right)}^{2}}{2{R}_{\infty }}\propto \frac{{\text{K}}}{{\text{S}}}\propto \alpha ,$$

where ${\text{F}}\left({R}_{\infty }\right)$ is the Kubelka − Munk function, ${R}_{\infty }$ is the diffuse reflectance ratio between the reference sample and measured one, ${R}_{\infty }={R}_{sample/{R}_{refrence}}$, S and K are respectively the scattering and the absorption Kubelka–Munk coefficients^44^. Absorbance spectra in spinel ferrites structure is related to the excitation of electrons from the valence band to the conduction band by photon radiation (O-2p ligand to Fe-3d metal level) or the 3d^5^-3d^4^ 4s^1^ transition in Fe^3+^ ions^19^. The band gap energy can be assessed by Tauc’s equation^29^:

23 $$\left({\text{F}}\left({R}_{\infty }\right)\mathrm{h\nu }\right)={\text{A}}{\left(\mathrm{h\upsilon }-{{\text{E}}}_{{\text{g}}}\right)}^{{\text{n}}}.$$

**Figure 10:**
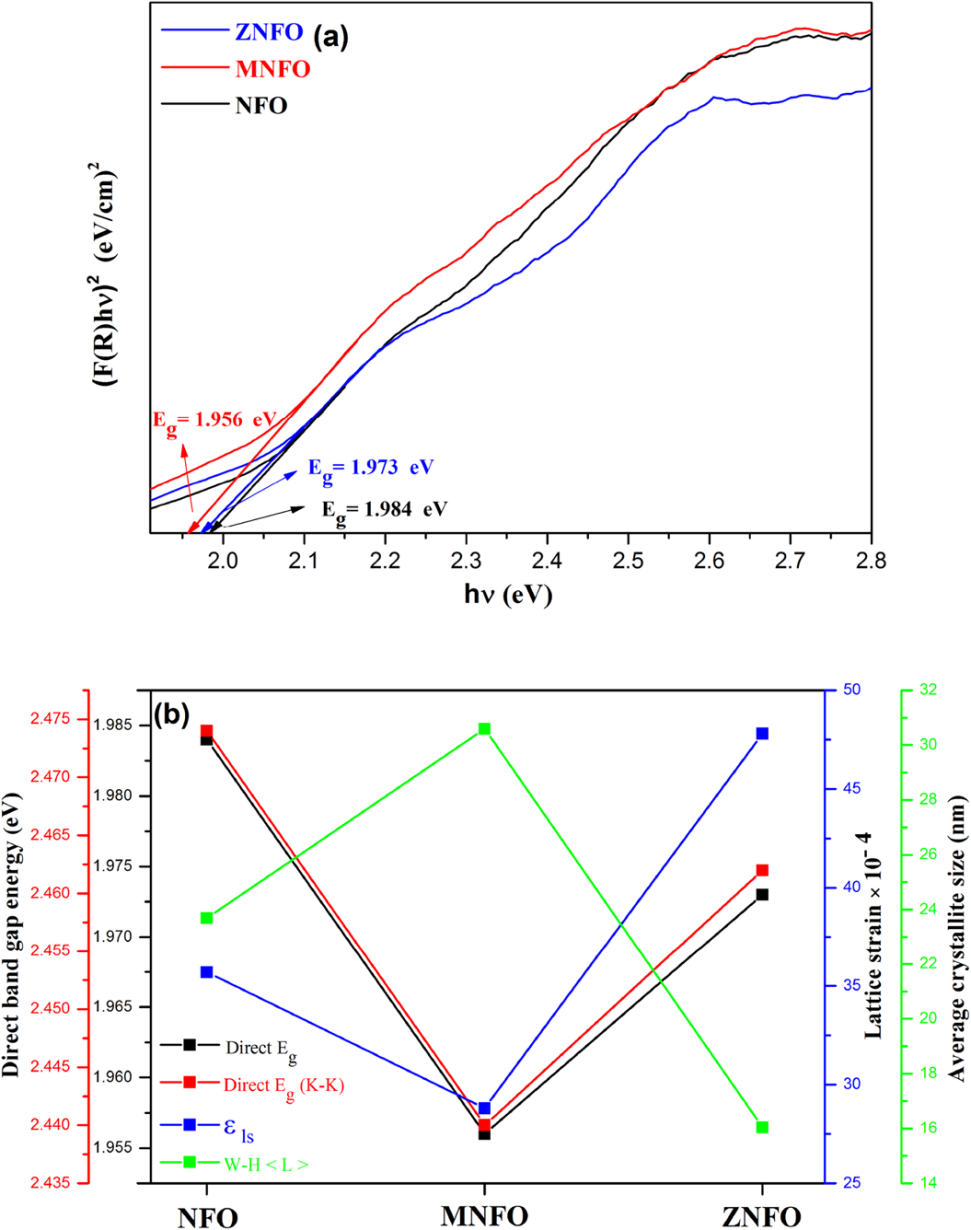
(**a**) Plot of (F(R)hν)^2^ against photon energy (hν), (**b**) comparison in alteration of direct band gap energy calculated by Kubelka–Munk function and Kramers–Kronig method with lattice strain (ε_ls_), and W–H average crystallite size < L > for all synthesized samples.

In which A is the proportionality constant that describes the degree of disorder in the sample, $\mathrm{h\nu }$ is the incident photon energy, and E_g_ is the band gap energy of the semiconductor. The value of n is related to the nature of electronic transition and can be the values of n = 1/2, 2, 3/2, or 3 that n = 1/2 is for direct allowed transition, and n = 2 is for indirect allowed transition^29^. The E_g_ can be estimated with the plotting of (F(R)$\mathrm{h\nu }$)^2^ in terms of the photon energy ($\mathrm{h\nu }$) and extrapolating the liner part of the plot for zero absorption spectra, (α $\mathrm{h\nu }$) = 0 as shown in Fig. 10a and represented in Table 6. As can be seen in Table 6, the E_g_ value of NFO was estimated at 1.984 which are comparable with those presented in the literature^1^. The E_g_ values of MNFO, and ZNFO MNPs were estimated at 1.956, and 1.973 eV, respectively. As shown, the bandgap energy of MNFO and ZNFO nanoparticles decreased comparing to NFO nanoparticles (redshift). K. Sasikumar and his coworkers estimated E_g_ of Mn-doped NiFe_2_O_4_ thin films to be around 3.3 eV in different Mn values (Ni_1-x_Mn_x_Fe_2_O_4_, *x* = 0, 3, 6, 9, and 12 wt%)^46^. In a study by C. Barathiraja, Mn doped Ni ferrite prepared by a facile microwave combustion method and found that E_g_ value was 2.11 eV for 10% Mn doped, which enhanced in compare to un-doped NiFe_2_O_4_ (2.02 eV)^47^. P.Annie Vinoshaa et al. synthesized Ni_1-x_Zn_x_Fe_2_O_4_ (x = 0.01, 0.03, 0.05, and 0.10) by co-precipitation route with NaOH reduction agent. They found E_g_ value has an enhanced trend in comparison to pure Ni ferrite and has E_g_ = 2.17 eV amount for 5% Zn doped NiFe_2_O_4_^37^. Compared to previous literature, in this study E_g_ values of Mn and Zn doping Ni ferrite decreased which may can be advantageous in photocatalytic activity^48^. In photocatalyst application, the bandgap energy must be in the range of 3.0 > E_g_ > 1.23 for visible light activity, so the narrow E_g_ values of NiFe_2_O_4_ and 5% doped Ni spinel ferrites can be helpful candidate for photocatalytic applications^49,50^. Furthermore, reduction of E_g_ value for doped samples can be suggest that Mn^2+^ and Zn^2+^ ions interred in the structure of NiFe_2_O_4_ spinel ferrite.

**Table 6:** Optical parameters of NFO, MNFO, and ZNFO spinel ferrites.

Samples	Direct band gap E_g_ (eV)		E_U_ (meV)
	Kubelka − Munk	Kramers–Kronig	
NFO	1.984	2.474	314
MNFO	1.956	2.440	409
ZNFO	1.973	2.462	368

The amount of the direct band gap appertained to many factors such as crystallite size, degree of structural disorder in the lattice, and presence of impurities^6^. According to XRD data in Mn-doped nickel ferrite, the crystallite size increased, so this can decrease the E_g_ value of MNPs because of the quantum confinement effect in semiconductor ferrites^48^, which is in accordance with estimated results as depicted in Fig. 10b. Also when the grain size is enhanced at a high annealing temperature (800 °C), as mentioned in TEM results, the grain boundaries are reduced, therefore, this decreases the scattering of carriers at grain boundaries, so that the E_g_ value can be diminished^51^. In Zn-doped NiFe_2_O_4_ despite the decrement in crystallite size of ZNFO from XRD data because of increment in the degree of the structural disorder means greater dislocation density, lattice strain, and micro-strain according to XRD results in Tables 4 and 5, the sub-bands formed and merged between the band gap which formed a continuous band that reduced the band gap energy of ZNFO spinel ferrite^52^ as shown in Fig. 10b. Also, the ionic radius can be the other reason for the band gap decrement of MNFO and ZNFO spinel ferrites. Ionic radiuses of Mn and Zn dopant cations are greater than Fe^3+^ and Ni^2+^ cations in tetrahedral and octahedral sites according to XRD results, hence the lattice constant increased, and in this condition, electrons constraint to the nucleus be more lose which expresses the electrons need less energy to leave the nucleus, therefore the band gap decrease and be narrow^53^.

Furthermore, the decrement of direct band gap energy can be ascribed to the sp-d exchange interactions between delocalized conduction band electrons and localized electrons in the d-shell of Mn^2+^ or Zn^2+^ ions that are substituted instead of Ni^2+^ ions. The exchange interactions of the Mn and Zn ions increased valence band potential and decreased conduction band potential, which decreased the band gap energy and a redshift happened^52^. So, it can be concluded that by Mn and Zn doping NiFe_2_O_4_, the band gap energy is reduced, which can be helpful in the improvement of optoelectronic applications^49^.

##### The absorption band tail (Urbach energy)

**Figure 11:**
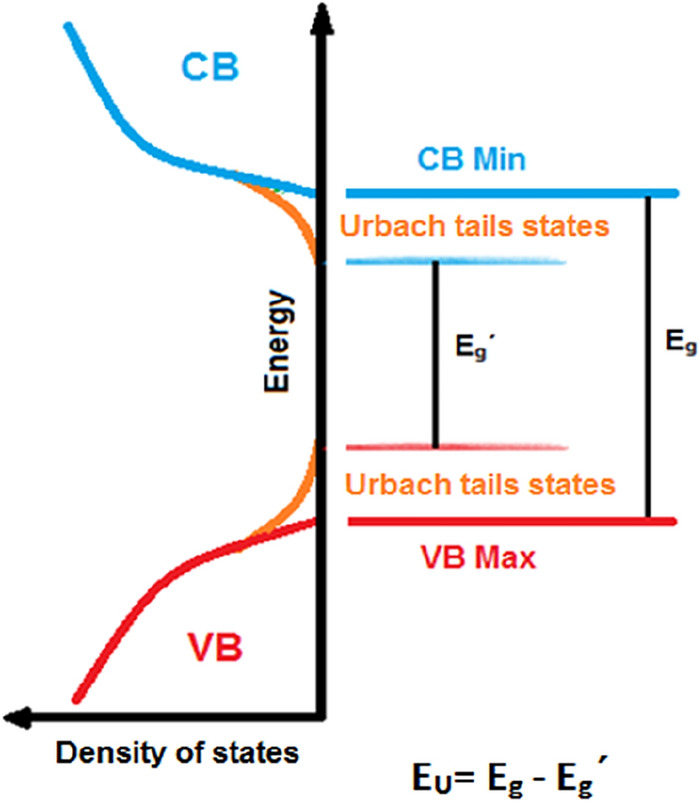
Schematic representation of Urbach tail states in a semiconductor system.

For further investigation on the pre-absorption edge and expounding the effects of defects and Mn and Zn doping on the crystal lattice of NiFe_2_O_4_, the Urbach energy (E_U_) is defined. The E_U_ is a crucial parameter for the estimation of the defects in material. These defects create absorption tails (localized states) between the conduction band and valence band in band states that named Urbach tails, and relative energy is called Urbach energy, as demonstrated in Fig. 11^54^. The Urbach energy can be calculated by using the following relations^54,55^:

24 $$\mathrm{\alpha }={\mathrm{\alpha }}_{0}{\text{exp}}\left(\frac{\mathrm{h\nu }}{{{\text{E}}}_{{\text{U}}}}\right),$$

25 $${\text{lnF}}\left({\text{R}}\right)=\mathrm{ln\beta }+\frac{\mathrm{h\nu }}{{{\text{E}}}_{{\text{U}}}} ,$$

where, α is the absorption coefficient (F(R) ≈ α), α_0_ is a constant, E = hυ is the incident photon energy, and $\upbeta =2{\mathrm{\alpha }}_{0}/S$ is a constant. The Urbach energy is determined by linear fitting of plotting ln(α) vs. (hν) and the reverse slope of the line as shown in the following equation:

26 $${E}_{U}={\left[\frac{d({\text{ln}}\left(\alpha \right))}{d(h\upsilon )}\right]}^{-1}.$$

**Figure 12:**
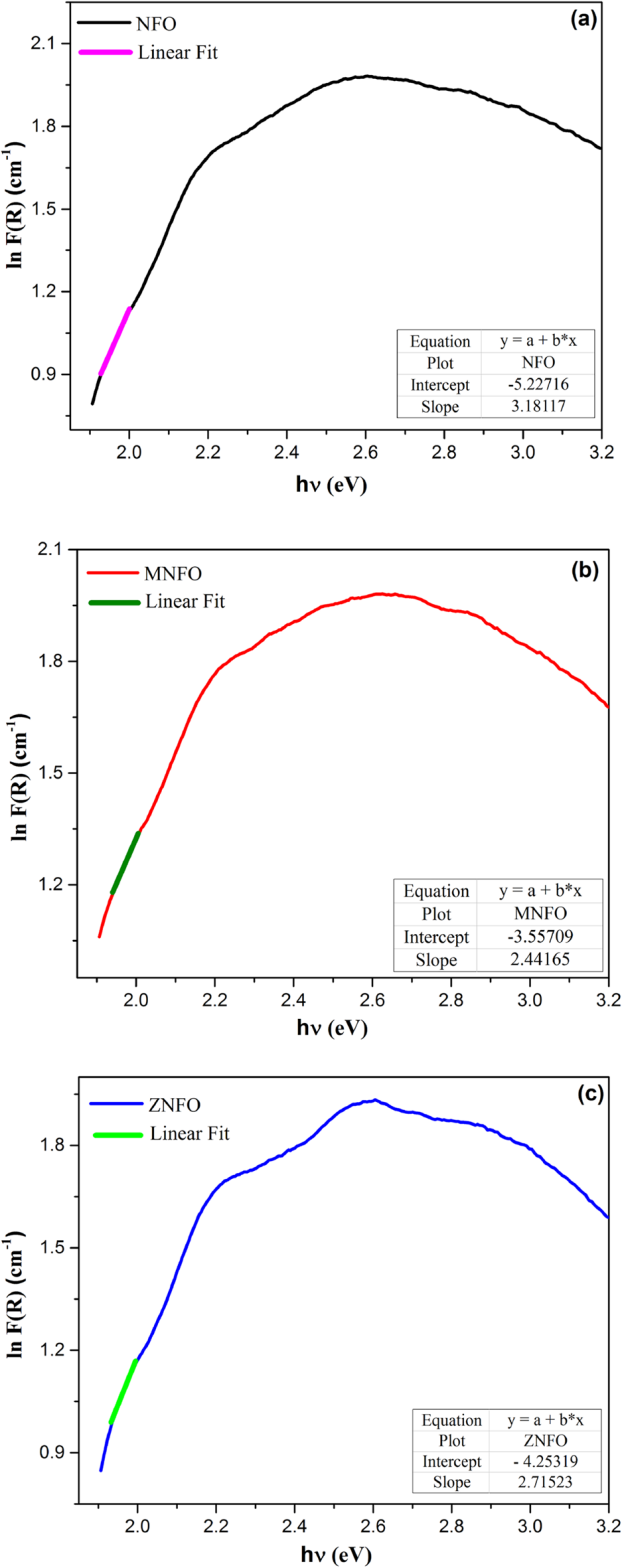
Plots of Urbach energy against photon energy (hν) for (**a**) NFO, (**b**) MNFO, (**c**) ZNFO.

Amounts of the Urbach tail energy of prepared samples are shown in Fig. 12 and tabulated in Table 6. The E_U_ values of all MNPs are almost low values that can imply a low degree of disorder and defects in the synthesized samples, which agree well with XRD results. The reason can be attributed to the high annealing temperature. Heat treatment causes the structure of the material to be organized, therefore, structural disorders of the material decrease^29^. Also, synthesized methods and conditions can be important in this characterization^56^. This condition often implies excellent electronic properties, such as high carrier mobility and low density of localized states^51,57^.

From Table 6, the E_U_ value increased (blue shift) with Mn and Zn substitution that proves, Mn^2+^ and Zn^2+^ dopant cations can systematically enhance the structural and electronic disorder in semiconductor Ni ferrites by a change in the bond angles and bond lengths in the lattice space of spinel ferrite. So, these factors can create localized states that increase the Urbach energy in the band gap region^57,58^. Also, when the impurity atom is introduced in the host lattice, the presence of the Mn^2+^ and Zn^2+^ dopant ions instead of some Fe^3+^ and Ni^2+^ sites in the tetrahedral and octahedral sublattice as explained in the XRD part, altered the Columbic potential of Ni^2+^ and Fe^3+^ sites because of difference in the ionic radii and atomic number (Z) therefor this effects in systematic scaling of the Urbach energy and organized the disordered states near to the valance and conduction bands^57^. From Table 6, the band gap energy decreased in contrast Urbach energy for doped samples which is accordance to the inverse relation between E_g_ and E_U_.

##### Linear optical study (Kramers–Kronig method)

In semiconductor materials, the refractive index complex is the key parameter in deciding the quality and nature of optoelectronic devices to plan hetero-structure lasers and in solar cell applications^19^. So, the accurate value of the refractive index and the calculation method to obtain it would be significant. Among numerical methods^59^, the Kramers–Kronig method is the more accurate and simple method that is utilized in the present study to determine the accurate real and imaginary parts of refractive index complex by DRS data to investigate linear optical properties, and are expressed by following relations^60,61^:

27 $$\mathrm{N^{\prime}}\left(\upomega \right)={\text{n}}\left(\omega \right)+{\text{ik}}\left(\upomega \right),$$

where n(ω) is the refractive index and k(ω) is the extinction coefficient. These coefficients were determined by the reflectance of a material as a function of polarization and the angle of incidence photon radiation, as given by the following equations^60,62^:

28 $${\text{n}}\left(\omega \right)=\frac{\left(1-{\text{R}}\left(\omega \right)\right)}{1+{\text{R}}\left(\omega \right)-2\sqrt{{\text{R}}\left(\omega \right)}\mathrm{cos\varphi }\left(\omega \right)},$$

29 $${\text{k}}\left(\omega \right)=\frac{2\sqrt{{\text{R}}\left(\omega \right)}\mathrm{sin\varphi }\left(\omega \right)}{1+{\text{R}}\left(\omega \right)-2\sqrt{{\text{R}}\left(\omega \right)}\mathrm{cos\varphi }\left(\omega \right)} ,$$

Here, ω is the angular frequency, $\mathrm{\varphi }\left(\omega \right)$ is the phase division of the reflected (${\text{R}}\left(\omega \right))$ and incident radiations, which is calculated by the Fourier transform and is expressed as follows^60^:

30 $$\varphi \left(\omega \right)=\frac{-\omega }{\pi }{\int }_{0}^{\infty }\frac{lnR\left(\omega^ {\prime}\right)-lnR(\omega )}{{\omega ^{\prime}}^{2}-{\omega }^{2}}d\omega ^{\prime} .$$

It is worth to note that all the calculation of this section (n, k) were done by MATLAB coding.

**Figure 13:**
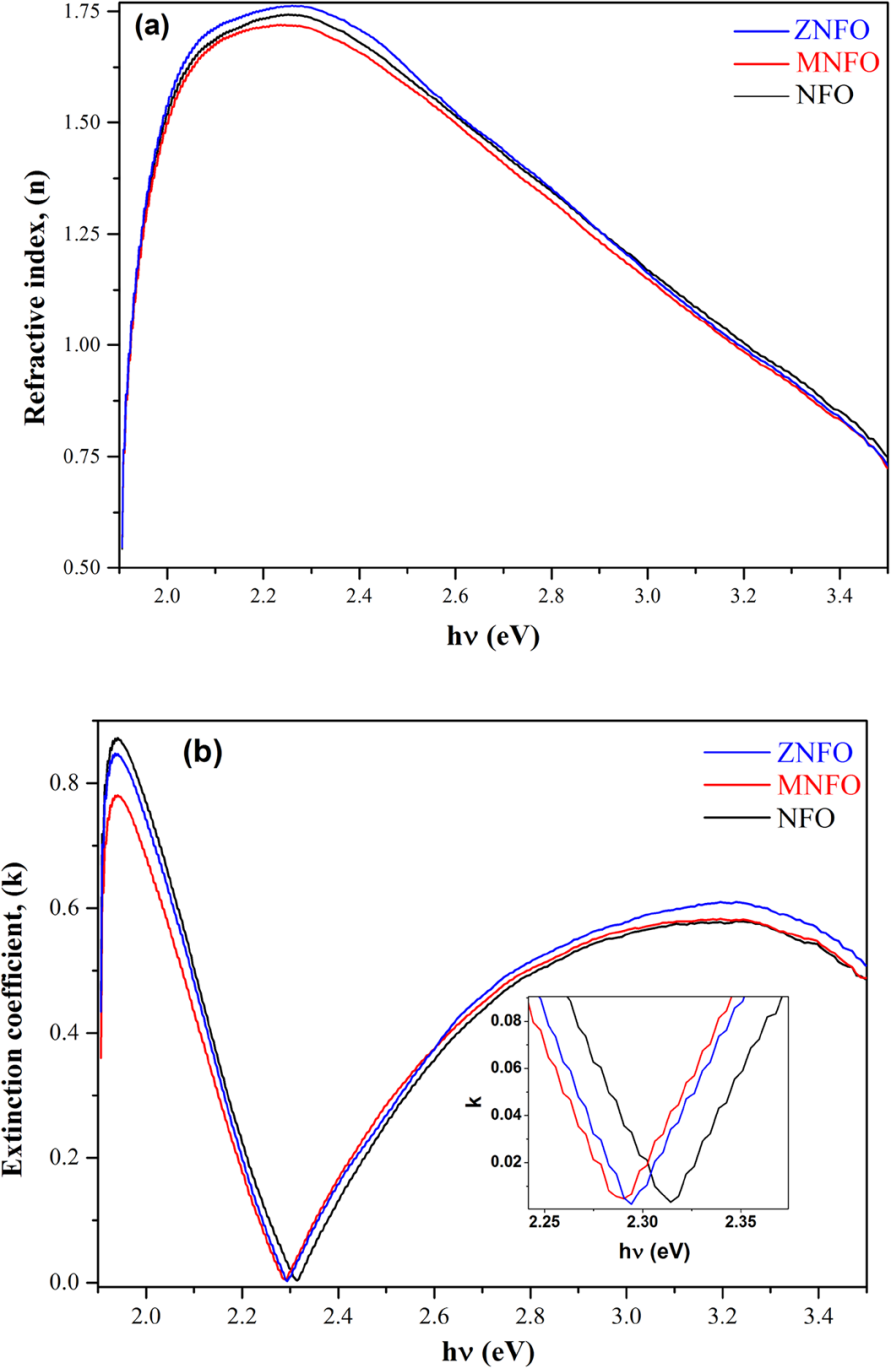
(**a**) The real part of the complex refractive index (n), (**b**) the imaginary part of the refractive index complex (k), and zoom-in pattern in the minimum point of extinction coefficient in synthesized samples.

*Refractive index* The refractive index is the critical parameter that indicates the relative velocity and the spread of refraction of light through the material^63^. Plots of refractive index n(ω) for synthesized MNPs as a function of hυ in the range of λ = 350–650 nm by Kramers–Kronig method were illustrated in Fig. 13a. As can be seen, at the beginning for NFO spinel ferrite, the amount of refractive index enhanced in the range of hυ = 1.9–2.3 eV (λ = 619–516 nm) and attain a refractive peak at the point of 1.75 eV (λ = 548.166 nm) in the UV–visible region that it can be due to normal dispersion law. The refractive index in the visible region is decisive for solar cells, photocatalytic, and optoelectronic devices^38,63^. By increasing incident photon energy and decreasing wavelength (λ = 516–350 nm) in the plot, the n index sharply decreases. As can be seen, the refractive index of the MNFO MNPs has a slight decrease in n value at the peak region compared to NFO. It can be assigned to the absorption of the basic band gap. The refractive curve of ZNFO MNPs demonstrates a slight increment in the peak region compared to NFO spinel ferrite, which probably can imply a decrement of porosity in Zn-doped nickel ferrite that causes an increase in the refractive index.

*Extinction coefficient* Figure 13b depicts the alterations of extinction coefficient (k) for synthesized nanopowders by Kramers–Kronig method. It is evident that there is one primary broad k value around 2 eV value and wavelength of 621 nm in the UV–visible region for NFO spinel ferrite. In other definitions, at this wavelength (621 nm) the fraction of light lost induced by photons absorption and scattering is maximum^64^. By the Mn and Zn doping nickel ferrite, the k value slightly decreased in the peak region. Also, in the k spectra, the wavelength point of doped nickel ferrite in minimum absorbance value (E = 2.3 eV, λ = 538 nm) slightly shifted to lower photon energy and higher wavelength. The maximum amounts of n and k are represented in Table 7.

**Table 7:** Maximum values of optical parameters for NFO, MNFO, and ZNFO spinel ferrites.

Samples	n max	λ (nm)	k max	λ (nm)	α max	λ (nm)
NFO	1.742	548.166	0.870	639.926	0.01908	379.38
MNFO	1.719	548.166	0.780	639.926	0.01908	379.38
ZNFO	1.763	548.166	0.847	639.926	0.0199	379.38

**Figure 14:**
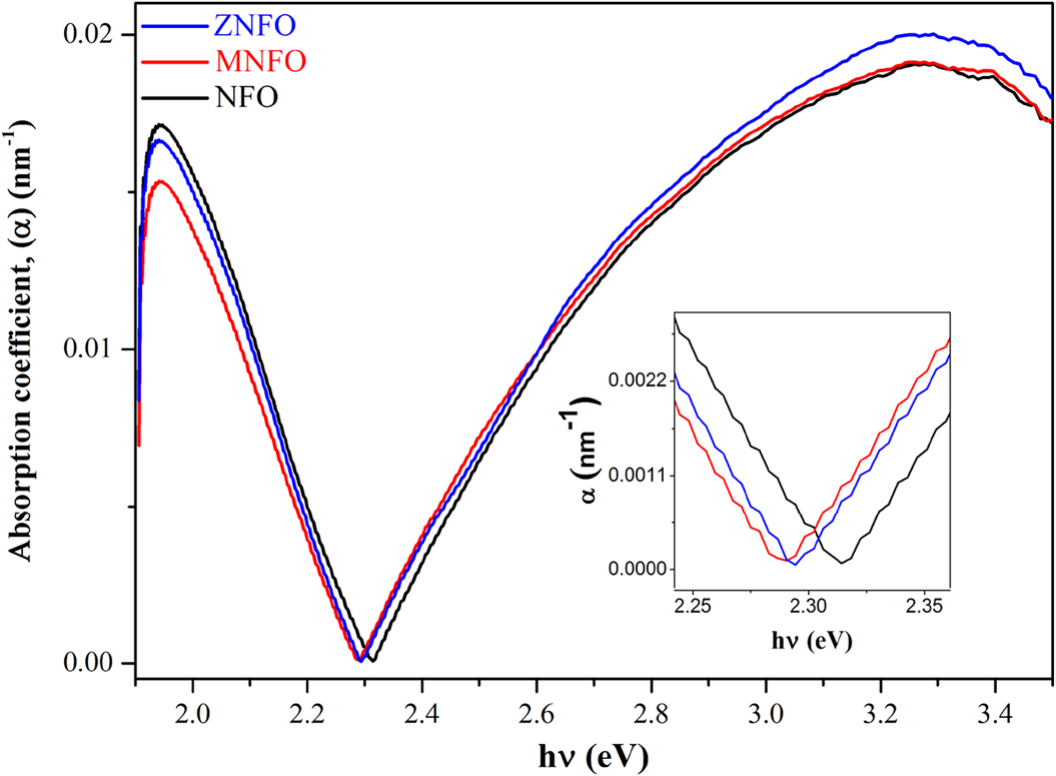
The absorption coefficient of synthesized samples and zoom-in patterns in the minimum point of the absorption coefficient.

**Figure 15:**
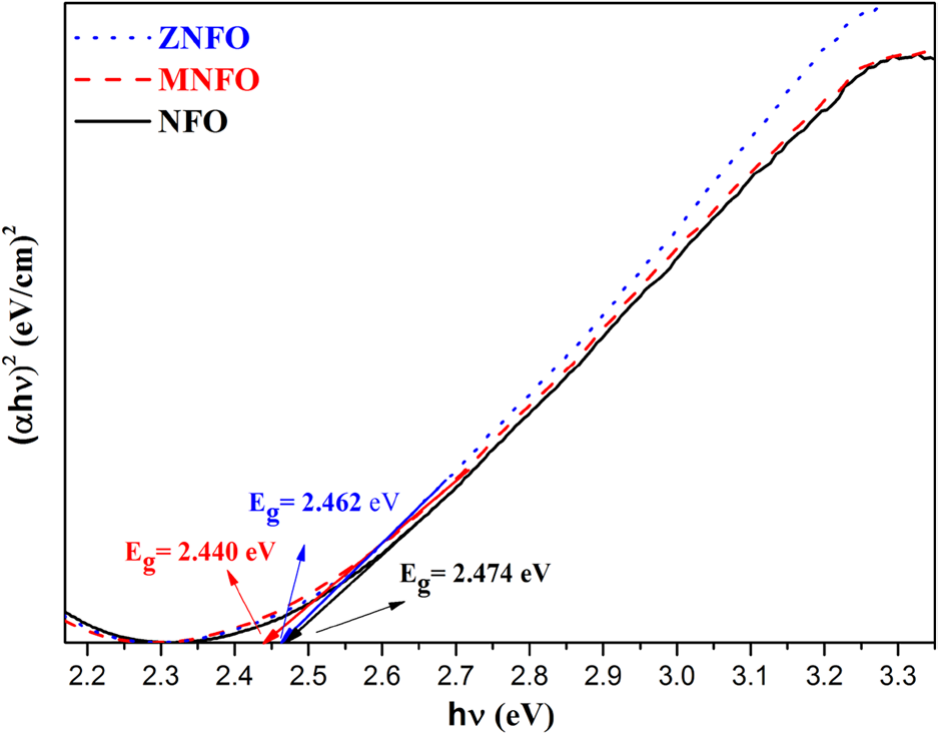
The band gap energy of synthesized nanoparticles by Kramers–Kronig method.

*Direct band gap (Kramers–Kronig method) *To the assessment of the band gap energy of the MNPs by the Kramers–Kronig (K-K) method, the absorption coefficient is utilized as follows:

31 $$\mathrm{\alpha }=\frac{4\mathrm{\pi k}}{\uplambda },$$

where k is the extinction coefficient calculated by K–K method (Sect. “Linear optical study (Kramers–Kronig method)”) and λ is the wavelength. Figure 14 depicts the absorption coefficient curve, and Table 7 represents the maximum value of α. The E_g_ of prepared specimens by K-K method was estimated using the absorption coefficient α instead of the F(R) in Eq. (23). The curve of band gap determination of un-doped and doped synthesized MNPs versus energy by K-K method is depicted in Fig. 15, and the estimated E_g_ values are tabulated in Table 6. From Fig. 15 and Table 6, it is evident that the E_g_ values calculated from the K-K method are in comparable accordance with the band gap values calculated by Kubelka–Munk function as depicted in Fig. 10b.

## Conclusions

In brief, we demonstrate that the co-precipitation technique with different material means hydrazine hydrate as reduction agent and ethylene glycol surfactant and heat treatment can provide an efficient way for the synthesis of M_x_Ni_1-x_Fe_2_O_4_ (M = Mn, Zn, and x = 0,0.05) spinel ferrites. The XRD spectra for the fcc structure of NFO, MNFO, and ZNFO, demonstrates that the samples have a high crystallinity without any molesting peak that can be due to the different material in synthesis process and heat treatment. The < L > , strain and other structural parameters were calculated by several methods and in comparison of methods, Williamson-Hall method is reasonable method without any restrictions. Band gap energies showed redshift by Mn^2+^ and Zn^2+^ ions substitution and the reduction trend of E_g_ in Mn and Zn-doped NiFe_2_O_4_ may can be useful in photocatalytic activity. The Urbach energy increased with a decrease in band gap energy and has an almost low value for NFO, MNFO, and ZNFO that indicates low structural disorder in accordance with XRD results. Estimated band gap energies by the Kramers–Kronig method are in good accordance with the Kubelka–Munk function method and can be a reasonable numerical method for band gap energy computing. By the K-K method, the refractive index illustrates that all samples have a maximum refractive index in the visible region.

## Data Availability

Data sets generated during the current study are available from the corresponding author on reasonable request.
